# Maintenance of demographic and hematological profiles in a long-lasting dengue fever outbreak: implications for management

**DOI:** 10.1186/s40249-016-0177-y

**Published:** 2016-09-05

**Authors:** Andréia Moreira dos Santos Carmo, Rodrigo Buzinaro Suzuki, Michele Marcondes Riquena, André Eterovic, Márcia Aparecida Sperança

**Affiliations:** 1 Center for Natural and Human Sciences, Universidade Federal do ABC, Campus São Bernardo do Campo, 09606-070 São Bernardo Do Campo, São Paulo Brasil; 2 Secretaria do Estado da Saúde do Estado de São Paulo, Instituto Adolfo Lutz, Centro de Laboratório Regional VIII, Santo André, 09040-160 São Paulo Brazil; 3 Discipline of Parasitology, Marilia Medical School, Marilia, 17519-030 São Paulo Brazil; 4 Center for Natural and Human Sciences, Universidade Federal do ABC, Campus Santo André, Santo André, 09210-170 São Paulo Brazil

**Keywords:** Clinical diagnosis, Demographic profile, Dengue fever, Dengue- outbreak pattern, Epidemiological threshold, Hematological profile

## Abstract

**Background:**

Dengue fever (DF) outbreaks present regionally specific epidemiological and clinical characteristics. In certain medium-sized cities (100 000–250 000 inhabitants) of São Paulo State, Brazil, and after reaching an incidence of 150 cases/100 000 inhabitants (“epidemiological threshold”), clinical diagnosis indicated dengue virus (DENV) infection. During this period, other seasonally infectious diseases with symptoms and physical signs mimicking DF can simultaneously occur, with the consequential overcrowding of health care facilities as the principal drawbacks. Confirmation of clinical diagnosis of DF with serological tests may help in avoiding faulty diagnosis in patients, who might later undergo dengue hemorrhagic fever (DHF) and the dengue-shock syndrome (DSS). Furthermore, demographic and hematological profiles of patients are useful in detecting specific early characteristics associated to DF, DHF and DSS.

**Methods:**

From March to June, 2007, 456 patients from Marilia in northwest São Paulo State who had only been diagnosed for DF by clinical criteria, underwent serologic testing for non-structural 1 (NS1) DENV antigens. Individual results were used in comparative analysis according to demographic (gender, age) and hematological (leukocyte and platelet counts, percentage of atypical lymphocytes) profiles. Temporal patterns were evaluated by subdividing data according to time of initial attendance, using recorded variables as predictors of DENV infection in logistic regression models and ROC curves.

**Results:**

Serologic DENV detection was positive in 70.6 % of the patients. Lower leukocyte and platelet counts were the most important factors in predicting DENV infection (respective medians DENV + = 3 715 cells/ml and DENV- = 6 760 cells/ml, and DENV + = 134 896 cells/ml and DENV- = 223 872 cells/ml). Furthermore, all demographic and hematological profiles presented a conservative temporal pattern throughout this long-lasting outbreak.

**Conclusions:**

As consistency throughout the epidemic facilitated defining the conservation pattern throughout the early stages, this was useful for improving management during the remaining period.

**Electronic supplementary material:**

The online version of this article (doi:10.1186/s40249-016-0177-y) contains supplementary material, which is available to authorized users.

## Multilingual abstracts

Please see Additional file 1 for translations of the abstract into the five official working languages of the United Nations.

## Background

The dengue virus of the genus *Flavivirus* (family Flaviviridae) is represented by four serotypes (DENV1 to 4), which are transmitted to humans by infected female *Aedes aegypti* and *A. albopictus* mosquitoes, widely distributed throughout tropical and subtropical regions [1]. According to the World Health Organization [2], 40 % of the population world-wide run the risk of DENV infection, with an estimated 50 to 100 million cases annually and around 20 000 fatalities.

Disease manifestation in DENV infected individuals ranges from absence or unspecified to classical dengue fever (DF), characterized by fever associated to muscle, joint and retro-orbital pain, photophobia and red body-rash. The most severe and lethal forms of the disease are characterized by increased vascular permeability, plasma leakage, thrombocytopenia and hemorrhagic manifestations [3, 4]. Risk factors, which vary in populations world-wide [5], include sequential DENV infection with a different virus serotype, diversity in genetic characteristics of both virus and host, vector dispersion and adaptability, and sequential virus serotype infection.

In medium-sized cities (100 000–300 000 inhabitants), DF outbreaks typically last for four to six months during the hot and rainy seasons, with specific epidemiological and clinical characteristics in space and time [6, 7]. In the New World, the spread of DENV has increased five-fold over the latter decades, with outbreaks occurring every three to five years through co-circulation of the four serotypes of DENV, with total severe dengue case fatality reaching 1.2 % [8]. Within this scenario and since 2000, Brazil has accounted for more than 70 % of DENV cases in the Americas, with a period of outstanding *A. aegypti* dispersion [9]. By mid-2007 and in all the dengue epidemics, adults from urban and surrounding locations were the most affected. However, in some regions and from the second half of 2007, there has been a change in this pattern, with a significant increase in severe dengue hemorrhagic cases in under-15-year-old children in the northeast and Rio de Janeiro State [10]. Nonetheless, the adult population continues to be the most affected. Hence, there is the need for regional studies to facilitate early recognition of any modification in the DENV epidemiological pattern, to thus improve control and treatment procedures.

In São Paulo State, concentration of DENV cases in urban centers has changed, with an increased incidence in small and medium-sized towns. In 2007, 52 % of the cases were reported in towns with less than 100 000 inhabitants, with 16 % occurrence in counties with populations ranging between 100 000 and 500 000 (Ministry of Health, 2007).

Marilia, a town in the west of São Paulo State, 450 km from the capital, with 216 745 inhabitants (IBGE 2010) and a large population flow due to economic relevance, represents a model for investigating DENV epidemiology in medium-sized towns state-wide. In the 2007 Marilia-outbreak, which reached 550 cases/100 000 inhabitants, in the first instance, only around 300 cases were confirmed through serological analysis. Subsequently, all suspected cases were required to be notified and submitted to hematological check-ups for detection and posterior treatment of severe hemorrhagic cases, whence the consequential overcrowding of health facilities.

During outbreaks, the use of such “epidemiological threshold” procedures in accordance with clinical criteria for DENV diagnosis, but without serological confirmation, imposes another significant problem; the co-occurrence of various seasonal infectious diseases with similar symptoms. These include other arboviruses, such as the flavivirus Saint Louis Encephalitis [11], Chikungunya fever [12, 13], Zika fever [14], leptospirosis [15, 16], visceral leishmaniasis [17], and malaria [18].

Certain demographic characteristics, such as the age of patients diagnosed with DENV, can be associated to distinct epidemiological patterns of DENV transmission [19]. Furthermore, early identification of the hematological profile during the fever period can differentiate DENV from other infectious diseases [20]. Thus, the aim hereby was to assess the demographic and hematological profiles of patients under treatment at the Marilia Hemocenter during the 2007 DF outbreak, and diagnosed by clinical criteria according to *DF epidemiological threshold* procedures. DENV infection in such individuals was investigated a *posteriori* (retrospectively) by way of serological testing.

## Methods

### Patients and laboratory analysis

When, during the 2007 outbreak, serologically confirmed dengue incidence reached the epidemiological threshold of 150/100 000 inhabitants in Marilia, 754 patients were diagnosed for dengue only by clinical means. They were attended to at the Marilia Hemocenter from March, 7 to June, 4. From the onset, they underwent a *posteriori* investigation to assess the serological status (DENV presence/absence), and demographic (gender and age) and hematological (leukocyte and platelet counts, and percentage of atypical lymphocytes) profiles of each. Only individuals with complete records (gender, age, leukocyte and platelet counts, and percentage of atypical lymphocytes) came under analysis. Hematocrit results, also monitored from the beginning, showed less than 6 % of investigated patients (26 out of 456) presented values above standard levels (42 % for under-12-year-old children, 44 % for adult women and 50 % for adult men) [21]. Percentages of red blood cells among under-12-year-olds (*n* = 34) ranged from 27.5 to 45.5 (median - 38.3; first and third quartiles 35.3 and 41.3, respectively); among women (*n* = 226) from 32.2 to 49.6 (median - 40.0; first and third quartiles 37.8 and 42.0, respectively); and among men (*n* = 196) from 29.1 to 49.9 (median - 44.1, first and third quartiles 41.5 and 45.8 respectively). However, since according to clinical and laboratory follow up, no patient presented hemoconcentration, these data were not included for further analysis. Blood-samples were collected in EDTA tubes from each patient for cell counting. The remaining material was centrifuged for plasma separation, and after frozen at −20 °C. Atypical lymphocytes were investigated by blood smear microscopic examination after Giemsa staining. Plasma samples underwent DENV serological diagnosis by enzyme linked immunosorbent assay detection of non-structural 1 (NS1) antigen, using the “Focus Diagnostics’ Dengue NS1 Antigen DxSelect™ Assay”, according to manufacturers’ instructions. Research protocol was approved by the Ethics Committee on Human Research of the Marilia Medical School (number 069/03).

### Statistical analysis

Among the 456 patients with complete records, and based on serological outcomes (positive cases = DENV+; negative cases = DENV-) two groups were described through distribution by gender (labeled as SEX, with males coded as 1, females as 0), age in years (AGE), leukocyte and platelet counts (respectively, LEU and PLA, both in Log_10_ cells/ml), and percentage of atypical lymphocytes (AL%). On the first day of attendance at the Hemocenter, DAY, counted from the start of the 89-days-interval, was also used in the survey. Within each group, these variables were evaluated separately by descriptive statistics. The groups were compared using the *X*^2^ test for SEX (in a 2×2 contingency table), and the Mann–Whitney for the remaining variables. Bivariate analysis was by Spearman rank correlations. In a contrary approach, the six variables were evaluated as independent explanatory factors, and according to their probability to predict DENV serological outcome. Logistic regression models were used to detect significant effects of each factor on the dichotomic response variable (DENV+ as 1, DENV- as 0). An additive model including all the six factors was also built. Parameters from the first set of models (explanatory factors used individually) described the relationship between each factor and the response variable by means of logit functions. Analysis was with the Pezzulo (2012) free online device for calculating logistic regression. Additional estimates of confidence intervals were obtained with Statistica 8.0 (Statsoft Co) software. Akaike Information Criteria (AIC values) were computed in an R software package. For all the variables except SEX, sigmoidal curves relating values to the response variable were plotted. Receiver operating characteristic (ROC) curves were plotted for all the factors except SEX. Confidence intervals for both specificity and sensibility were compiled with the R package pROC [22], as was the case of comparison among areas below the curves (AUC).

We evaluated how the different subsets of patients obtained from distinct periods of first attendance at the Marilia Hemocenter could generate particular individual explanations of serological outcome with the selected factors. Data were divided into four subsets, each comprised of an equal number of individuals (s1 to s4; *N* = 114 in all), with the first and the last quarter of patients to arrive at the Marilia Hemocenter being placed accordingly in the subsets. Each of the four was treated as an entire set of data to so build up new logistic regression and ROC curves. Comparison was between these subsets and the entire sample. Unless otherwise explicitly stated, *P*-values were considered following Bonferroni correction based on the number of similar tests.

## Results

As an indication of the absence of sampling bias, data from the discarded group of patients with incomplete records (N_out_ = 330) did not differ significantly from those of the group of patients included in the analysis (N_in_ = 456), when considering (a) sex (2×2 contingency table: *X*^2^ = 35.4; *P* = 0.3775; N_out_ = 330), (b) age (Mann–Whitney U = 28 940; *P* = 0.1907; N_out_ = 137), (c) day of the first attendance at the Hemocenter (Mann–Whitney U = 73 110; *P* = 0.4982; N_out_ = 330), or (d) representativeness of original location (number of patients in both groups from 23 Marilia districts compared by Spearman rank correlation: rS = 0.826; *P* < 0.0001; N_out_ = 82).

A summary of variables and univariate comparison between the two groups of serological outcome appears in Table 1. Among the 456 patients evaluated, 322 (70.6 %) were serum positive (DENV+). In the latter, 48.1 % were males with median age of 35 years. The median time of the first attendance at the Marilia Hemocenter since sampling started was 41 days. Among serum-negative patients (DENV-), males comprised 42.6 % with a median age of 29 years. The Median time from first attendance was 38.5 days. For the three variables SEX, AGE, and DAY, differences among groups were insignificant. Groups differed significantly as regards hematological conditions. Leukocyte and platelet counts were lower in positive patients (leukocyte medians: DENV + = 3 715 cells/ml and DENV- = 6 760 cells/ml; platelet medians: DENV + = 134 896 cells/ml and DENV- = 223 872 cells/ml) Transformed logarithmic values are presented in Table 1. Percentages of atypical lymphocytes were lower among negative patients (medians: DENV + = 4 % and DENV- = 1 %). In both groups, hematological variables were mutually strongly correlated. Whereas in leukocyte and platelet counts this was positive, in atypical lymphocytes it was negative (Table 2). With the exception of DAY and platelet counts in DENV-patients, there were no correlations between the variables SEX, DAY and AGE or any other variable in any group.

**Table 1:** Demographic and hematological status of DENV+ and DENV- patients in the 2007 Marilia outbreak

Variable		DENV+	DENV-	Test	*P*
SEX	% males	48.1	42.5	1.19	0.2748
DAY	Mean	42.25	37.84	19,050	0.0485
	Standard deviation	19.906	20.396		
	Median	41	38.5		
	Range	(0–86)	(0–86)		
AGE	Mean	37.08	32.55	18,410	0.0137
	Standard deviation	18.558	19.774		
	Median	35	29		
	Range	(0–85)	(1–78)		
LEU	Mean	3.57	3.83	7654	**<0.0001**
	Standard deviation	0.205	0.197		
	Median	3.56	3.83		
	Range	(2.95–4.30)	(3.28–4.39)		
PLA	Mean	5.12	5.33	8830	**<0.0001**
	Standard deviation	0.214	0.180		
	Median	5.13	5.35		
	Range	(4.00–5.69)	(4.64–5.87)		
AL%	Mean	6.52	2.50	13,160	**<0.0001**
	Standard deviation	6.791	3545		
	Median	4	1		
	Range	(0–39)	(0–20)		

Demographic and hematological status of patients from two groups based on serological outcomes in Dengue virus testing (DENV+ *N* = 322, DENV- *N* = 134). Gender (SEX), age (AGE, in years), leukocyte and platelet counts (respectively LEU and PLA, both in Log_10_ cells/ml), percentage of atypical lymphocytes (AL%) and day of the first attendance at the Marilia Hemocenter (DAY, counted from March 07, 2007, when sampling started) were recorded for 456 patients. All test values are Mann–Whitney U comparisons among groups, except for SEX (*X* ^2^ applied to a two-way contingency table). *P*-values lower than critical (*P*_c_ = 0.05/6 = 0.0083, considering six contrasts in a Bonferroni correction) were shown in bold

**Table 2:** Spearman rank correlation among demographic and hematological variables for DENV+ and DENV- patients

	DENV+		DENV-	
X x Y	Rs	*P*	Rs	*P*
SEX x DAY	−0.023	0.6847	−0.078	0.3696
SEX x AGE	−0.093	0.0971	−0.017	0.8421
SEX x LEU	0.012	0.8287	−0.142	0.1020
SEX x PLA	−0.083	0.1379	−0.185	0.0326
SEX x AL%	0.026	0.6422	0.112	0.1985
DAY x AGE	0.733	0.0191	0.202	0.1110
DAY x LEU	−0.016	0.7739	−0.171	0.0488
DAY x PLA	0.062	0.2679	−0.299	**0.0004**
DAY x AL%	0.139	0.0124	0.104	0.2303
AGE x LEU	0.094	0.0915	−0.061	0.4872
AGE x PLA	0.030	0.5974	−0.172	0.0475
AGE x AL%	−0.038	0.4954	0.045	0.6053
LEU x PLA	0.496	**<0.0001**	0.336	**0.0001**
LEU x AL%	−0.318	**<0.0001**	−0.376	**<0.0001**
PLA x AL%	−0.482	**<0.0001**	−0.274	**0.0014**

Spearman rank correlation (R_S_, and their respective *P*-values) among demographic and hematological variables in two groups of patients (DENV+ *N* = 322, DENV- *N* = 134). Figures lower than critical *P* were in bold. For 15 correlation tests in each group of patients, critical *P* = 0.05/15 = 0.0033. Variable codes as in Table 1

**Fig. 1:**
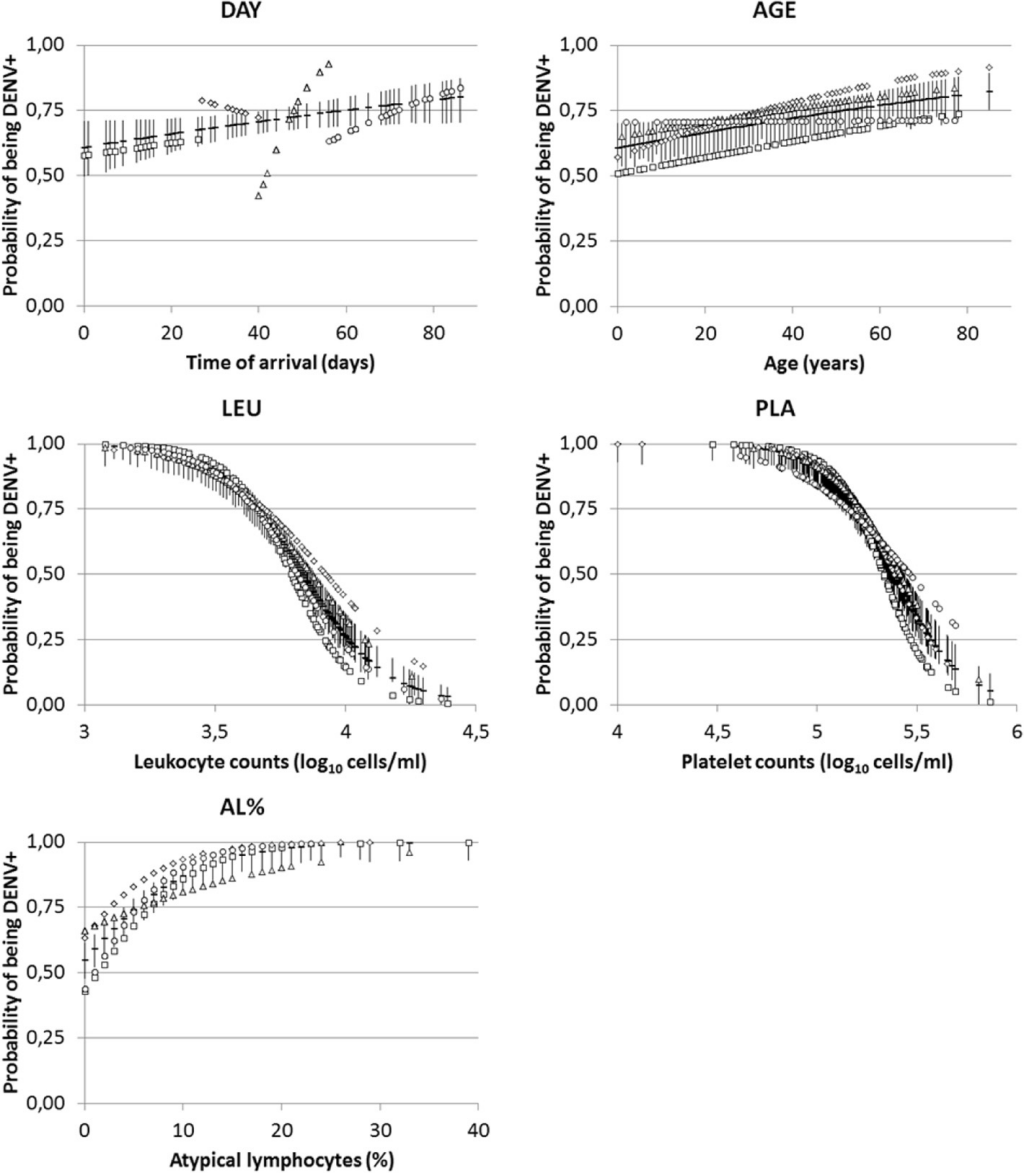
Sigmoidal curves showing individual contribution of demographic and hematological factors as independent predictors of DENV infection, based on parameters of logistic regression in Table 2. Horizontal bars represent the entire dataset (with associated 95 % confidence intervals). Subsets (see text) are s1 = squares, s2 = diamonds, s3 = triangles, s4 = circles. Variable codes as in Table 1

Logistic regression showed significant LEU and PLA usefulness in foreseeing DENV serological outcome, as reinforced by models with individually presented factors for the entire dataset (Table 3). As already mentioned, these two variables were strongly correlated in both groups (DENV+ and DENV-; Table 2). The contribution of each factor (except SEX) as DENV-infection predictors was presented in sigmoidal curves (Fig. 1). When using the entire dataset, SEX, DAY and AGE proved to be non-significant factors (in the latter two, only proven after Bonferroni correction for critical *P*) in their respective and exclusive models. Among all the subsets, LEU and PLA alone remained individually significant factors. AL% (s1 and s4) and DAY (s3) also appeared punctually in the subsets as significant individual factors.

**Table 3:** Logistic regression models of demographic and hematological factors as individual predictors of DENV infection

		Dataset				
Factors	Statistics	All	s1	s2	s3	s4
Sex	β_o_	0.774	0.392	1.276	0.928	0.649
	β_1_	0.226	0.160	−0.288	0.325	0.660
	OR	1.254	1.173	0.750	1.384	1.934
	CL-	0.8352	0.5494	0.3175	0.5899	0.8172
	CL+	1.8823	2.5068	1.7716	3.2458	4.5777
	P	0.2752	0.6794	0.5119	0.4553	0.1335
	AIC	551.1	155.9	130.7	132.7	138.9
Day	β_o_	0.435	0.312	2.048	−7.409	−1.499
	β_1_	0.011	0.010	−0.027	0.178	0.036
	OR	1.011	1.010	0.973	1.194	1.037
	CL-	1.0009	0.9635	0.8820	1.0682	0.9876
	CL+	1.0214	1.0593	1.0737	1.3350	1.0891
	P	0.0334	0.6728	0.5868	**0.0018**	0.1443
	AIC	547.7	155.9	130.8	122.2	138.9
Age	β_o_	0.427	0.033	0.292	0.615	0.879
	β_1_	0.013	0.013	0.025	0.013	0.001
	OR	1.013	1.013	1.025	1.014	1.001
	CL-	1.0020	0.9934	0.9999	0.9896	0.9789
	CL+	1.0242	1.0331	1.0503	1.0379	1.0226
	P	0.0210	0.1948	0.0510	0.2717	0.9628
	AIC	546.8	154.3	126.9	132.1	141.2
LEU	β_o_	24.173	32.162	18.422	20.852	25.583
	β_1_	−6.3036	−8.4818	−4.6950	−5.3871	−6.7062
	OR	0.002	0.001	0.009	0.005	0.001
	CL-	0.0005	0.0000	0.0009	0.0004	0.0001
	CL+	0.0069	0.0048	0.0972	0.0594	0.0206
	P	**<0.0001**	**<0.0001**	**<0.0001**	**<0.0001**	**<0.0001**
	AIC	440.6	99.9	111.3	110.7	107.4
PLA	β_o_	31.916	43.426	34.360	30.579	19.594
	β_1_	−5.931	−8.143	−6.377	−5.646	−3.586
	OR	0.003	0.001	0.002	0.003	0.028
	CL-	0.0007	0.0001	0.0001	0.0002	0.0027
	CL+	0.0108	0.0068	0.0359	0.0705	0.2855
	P	**<0.0001**	**<0.0001**	**<0.0001**	**0.0002**	**0.0026**
	AIC	464.6	109.8	106.7	115.1	130.3
AL%	β_o_	0.192	−0.287	0.547	0.672	−0.242
	β_1_	0.169	0.209	0.207	0.077	0.251
	OR	1.184	1.233	1.230	1.080	1.285
	CL-	1.1190	1.0950	1.0480	0.9891	1.1274
	CL+	1.2538	1.3875	1.4439	1.1793	1.4654
	P	**<0.0001**	**0.0005**	0.0113	0.0860	**0.0002**
	AIC	511.8	136.1	118.9	129.8	118.1

Logistic regression models to evaluate the individual contribution of demographic and hematological factors to DENV incidence using different datasets (ALL = entire data; subsets s1 to s4 ordered by day of the first attendance at the Marilia Hemocenter; see text). β_o_ = intercept, β_1_ = slope, *OR* odds ratio, and *CL* 95 % confidence limits. Figures lower than critical P were in bold. Considering six models (one for each factor) with each dataset, critical *P* = 0.05/6 = 0.0083. AIC indicate model fit (comparable both between subsets and factors for subsets s1 to s4; comparable between factors for ALL). Variable codes as in Table 1

In the all-inclusive additive logistic regression model for evaluating the contribution of the six DENV-infection explanatory factors (Table 4), only LEU and PLA presented significant effects in the entire dataset. LEU presented significant results in all the subsets (only this in s1, and with DAY in s3 and AL% in s4), except in s2. In this, and as AGE and PLA had been discarded by Bonferroni correction, no factor could be considered significant. In no subset did SEX, AGE and PLA appear as significant factors. A model using only LEU and PLA additively for the entire dataset resulted in an AIC value (409.4) lower than those of both “individualized” models (423.6 for LEU and 457.2 for PLA; Table 3). This two-factor model was slightly better than the “full” additive model (AIC = 412.1) with all the six explanatory variables (Table 4).

**Table 4:** All inclusive, additive logistic regression models of demographic and hematological factors as predictors of DENV infection

		Dataset				
	Statistics	ALL	s1	s2	s3	s4
	AIC	412.1	97.5	107.1	103.4	98.1
Factors	β_o_	30.194	54.258	30.300	31.291	15.871
Sex	β_1_	−0.007	−1.006	0.1092	0.3120	0.7480
	OR	0.993	0.366	1.115	1.366	2.113
	CL-	0.6035	0.1079	0.3908	0.4545	0.6700
	CL+	1.6342	1.2385	3.1827	4.1066	6.6626
	*P*	0.9784	0.1060	0.8383	0.5785	0.2018
Day	β_1_	0.002	−0.050	−0.007	0.197	0.073
	OR	1.002	0.951	0.993	1.218	1.076
	CL-	0.9900	0.8872	0.8854	1.0630	1.0049
	CL+	1.0145	1.0197	1.1132	1.3953	1.1520
	*P*	0.7310	0.1584	0.9012	**0.0045**	0.0356
Age	β_1_	0.011	0.006	0.035	0.018	−0.028
	OR	1.011	1.006	1.036	1.018	0.973
	CL-	0.9985	0.9786	1.0055	0.9887	0.9426
	CL+	1.0245	1.0339	1.0666	1.0488	1.0038
	*P*	0.0831	0.6755	0.0202	0.2274	0.0847
LEU	β_1_	−4.518	−6.303	−2.310	−4.632	−6.838
	OR	0.011	0.002	0.099	0.010	0.001
	CL-	0.0025	0.0000	0.0049	0.0004	0.0000
	CL+	0.0477	0.0840	20.160	0.2510	0.0430
	*P*	**<0.0001**	**0.0012**	0.1327	**0.0052**	**0.0003**
PLA	β_1_	−2.536	−5.556	−4.194	−4.392	0.947
	OR	0.079	0.004	0.015	0.012	2.578
	CL-	0.0150	0.0000	0.0003	0.0003	0.1099
	CL+	0.4192	0.3552	0.7826	0.5545	60.4591
	*P*	**0.0029**	0.0160	0.0374	0.0236	0.5564
AL%	β_1_	0.040	0.034	0.073	−0.090	0.262
	OR	1.041	1.035	1.076	0.914	1.299
	CL-	0.9828	0.9227	0.9101	0.8157	1.104
	CL+	1.1029	1.1603	1.2724	1.0245	1.529
	*P*	0.1703	0.5592	0.3909	0.1227	**0.0017**

All-inclusive, additive logistic regression models to evaluate the contribution of demographic and hematological factors to DENV incidence using different datasets (ALL = entire data; subsets s1 to s4 ordered by day of the first attendance at the Marilia Hemocenter; see text). AIC indicate model fit (comparable only between subsets s1 to s4). β_o_ = intercept, β_1_ = slope, OR = odds ratio, and CL = 95 % confidence limits. Figures lower than critical P were in bold. Considering five models (one for each dataset), critical *P* = 0.05/5 = 0.01. Variable codes as in Table 1

**Fig. 2:**
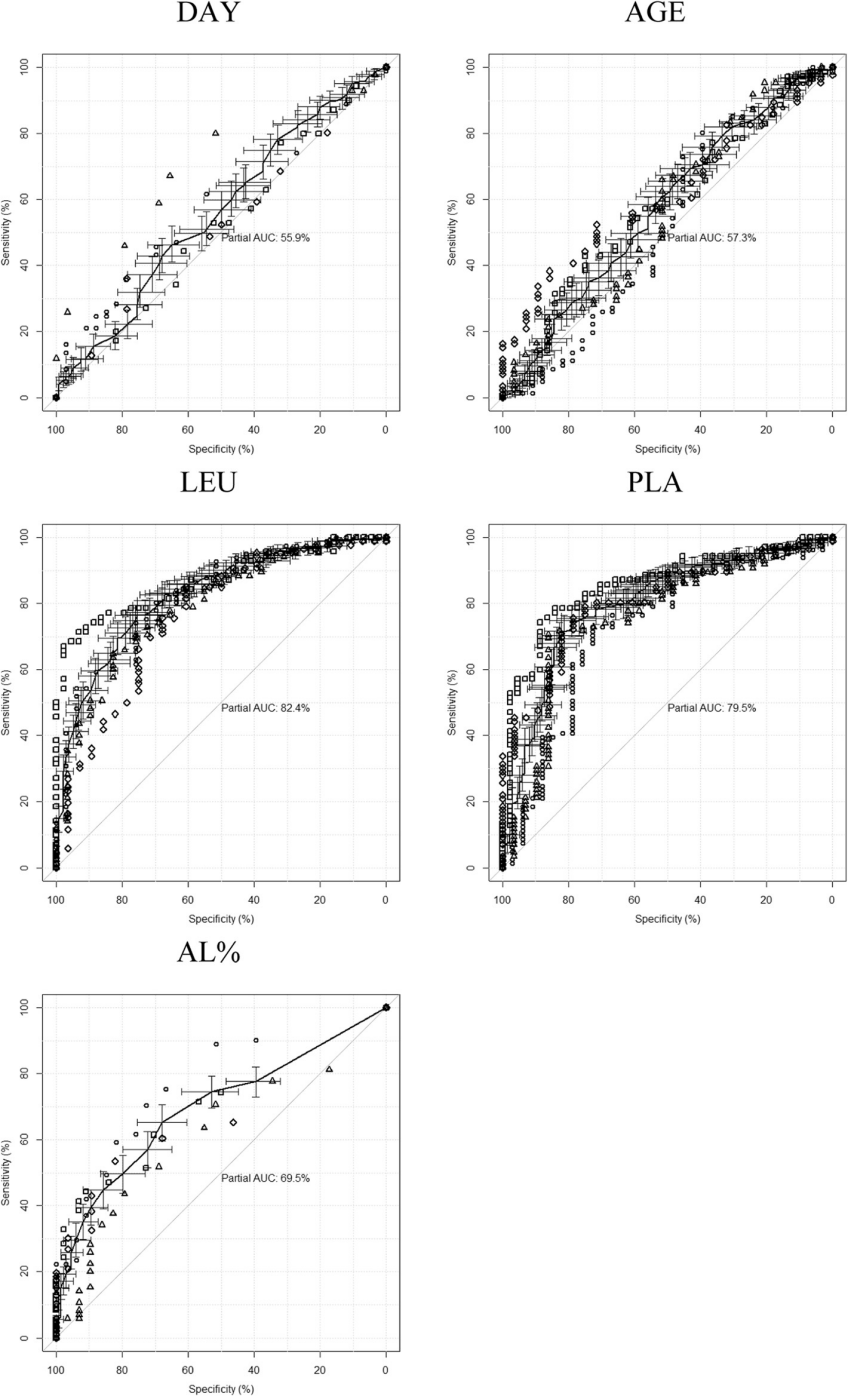
Receiver Operating Characteristic (ROC) curves showing the individual contribution of demographic and hematological factors as independent predictors of DENV infection, based on specificity and sensitivity. Black curves represent the entire dataset (with associated 95 % confidence intervals), for which their partial areas under curves (AUC) are presented. Lines represent the entire dataset (with associated 95 % confidence intervals). Subsets (see text) are s1 = squares, s2 = diamonds, s3 = triangles, s4 = circles. Variable codes as in Table 1

According to both sensitivity and specificity, the factors AGE and DAY differed slightly from chance in DENV discrimination: their ROC curves appear near the graph diagonal (Fig. 2, Table 5). LEU and PLA, in this order, were considered the best factors by simultaneously reaching the highest scores of true positives and true negatives, as indicated by high AUC values (Fig. 2, Table 5). The AL% was placed intermediately between the two groups of variables (Fig. 2, Table 5). Except for s3 in DAY, ROC curves computed for each factor of the subsets were not statistically different from those compiled for the entire dataset (Table 6).

**Table 5:** Paired comparison among Receiver Operating Characteristic (ROC) curves among demographic and hematological factors as predictors of DENV infection

Factor A	Factor B	AUC_A_	AUC_B_	D	*P*
LEU	PLA	82.41 (77.08–85.88)	79.54 (71.72–81.84)	1.88	0.0595
LEU	AL%		69.51 (65.63–77.56)	3.07	**0.0021**
LEU	AGE		57.32 (51.47–62.99)	6.77	**<0.0001**
LEU	DAY		55.86 (51.21–62.45)	7.28	**<0.0001**
PLA	AL%			1.45	0.1462
PLA	AGE			5.52	**<0.0001**
PLA	DAY			5.56	**<0.0001**
AL%	AGE			3.57	**0.0004**
AL%	DAY			3.80	**0.0001**
AGE	DAY			0.03	0.9733

AUC is area under the curve (%) and D is the statistics related to the difference between curves. *P* values lower than critical were in bold. Considering ten contrasts, critical *P* = 0.05/10 = 0.005. Factors appear in decreasing order of AUC, showed only at first time with respective 95 % confidence limits in parenthesis. Variable codes as in Table 1

**Table 6:** Paired comparison among Receiver Operating Characteristic (ROC) curves between subsets and the entire data for demographic and hematological factors as predictors of DENV infection

		Datasets				
Factor	Statistics	ALL	s1	s2	s3	s4
Day	AUC	55.86	51.61	52.49	70.24	57.95
	D		0.65	0.52	−2.41	−0.32
	*P*		0.5148	0.5970	**0.0160**	0.7449
Age	AUC	57.32	57.60	61.57	56.47	52.19
	D		−0.04	−0.67	0.12	0.69
	*P*		0.9655	0.5043	0.9040	0.4873
LEU	AUC	82.41	86.82	76.26	78.97	82.25
	D		−1.16	1.06	0.67	0.03
	*P*		0.2474	0.2894	0.5041	0.9726
PLA	AUC	79.54	85.57	80.67	75.80	72.45
	D		−1.38	−0.23	0.64	1.21
	*P*		0.1687	0.8152	0.5209	0.2269
AL%	AUC	69.51	70.67	66.34	61.87	77.61
	D		−0.22	0.59	1.18	−1.55
	*P*		0.8234	0.5574	0.2365	0.1222

AUC is area under the curve (%) and D is the statistics related to the difference between curves. All *P* values are above the critical (critical *P* = 0.05/4 = 0.0125, considering four contrasts for each factor). The only significant figure *before* such correction is in bold. Variable codes as in Table 1

## Discussion

Clinical criteria for post-epidemiological threshold phase DF diagnosis have been largely employed in developing countries, due to the technical and economic difficulties in carrying out specific tests to confirm DENV infection during outbreaks [23]. In these cases, blood-cell counts of patients with DF symptoms are the procedure in healthcare institutions, as a way of monitoring disease evolution with the purpose of preventing and treating the severe hemorrhagic form [24, 25]. Potentially, clinical signs and the hematological profile of patients with acute DENV infection can vary individually and regionally, due to epidemiological characteristics, such as the presence of other seasonal infectious diseases, host and virus genetic background, and previous virus circulation, thereby making the use of only clinical criteria for diagnosis questionable [26]. In order to investigate the effectiveness of such criteria applied to febrile patients at the Marilia Hemocenter during the 2007 outbreak, demographic and hematological status were assessed by applying *a posteriori* NS1-based DENV serological outcome data.

Through presenting high sensitivity and specificity in studies carried out in Brazil, NS1 antigen detection has proved to be an important tool in early DF diagnosis [27–30]. The kit employed in this investigation has an expected Positive Percent Agreement (PPA) of 88.2 % (95 % Confidence Interval: 73.4–95.3 %) and a Negative Percent Agreement (NPA) of 100 % (95%*CI*: 93.2–100.0 %) (manufacturer’s information). Accordingly, clinical criteria success was confirmed in 70.6 % of the cases reported. The remainder (29.4 %) was comprised of samples with a false DENV outcome or which corresponded to other infectious diseases. In order to increase the accuracy of DENV NS1 serological diagnosis, the detection of IgM/IgG DENV NS1 antibodies is recommended [31, 32]. However it was impossible to do so in the present study. Furthermore, serological tests to check the presence of the different infectious agents were omitted here.

Even though indicator efficiency of hematological changes during progression in distinguishing DENV from other infectious diseases is controversial, it remains essential in the clinical management of DF and severe hemorrhagic dengue [25, 33]. Leukocyte and platelet counts below 3 760 cell/ml and 100 000 cells/ml, respectively, and the presence of atypical lymphocytes, are commonly found in classical and severe cases of DF [20, 34, 35]. Our data showed a strongly significant association of decreased leukocyte and platelet counts in DENV+ patients. ROC curves reinforced LEU and PLA as the best factors for predicting serological DENV outcomes (Fig. 1).

Biochemical liver damage markers, such as aspartate aminotransferase (AST), and serum glutamic oxaloacetic transaminase (SGOT), as well as alanine aminotransferase (ALT) and serum glutamic pyruvic transaminase (SGPT) serum levels, are used to evaluate dengue disease severity [2]. However, in our health system these parameters are absent from the normal hematological exam routine, and thus could not be included in the study.

Data of the demographic status of a population involved in a DENV outbreak facilitates characterization of the epidemiological pattern of virus transmission, thereby contributing towards prevention and management. In Asia, where the number of severe hemorrhagic dengue cases is the highest in the world, with a fatality rate of 3.5 %, prevalence is higher in children under-15 [36]. In the 2007 Marilia DENV outbreak, the adult population was mostly affected by DF, with only five cases of the non-lethal hemorrhagic form of the disease (0.4 %). No differences in gender or age were found among DENV+ and DENV- patients. This DENV transmission pattern is the most common in the Americas [9].

In the different regions of Brazil, the time span of DF outbreaks after the establishment of an epidemiological threshold, is long-lasting, and can continue throughout the hot rainy season. In São Paulo State it can last for four to six months [37]. During this period, health care facilities are overcrowded and there is confusion in diagnosis with seasonal diseases. Moreover, and depending on the course of infection, it is possible that hematologic changes, so commonly associated with the more severe DENV diseases are not very evident in initial data. Thus, in order to further adapt monitoring of the disease, all patients suspected of DENV infection are assayed for hematological changes [38]. By taking into consideration the regional demographic and hematological profiles of DF and its more severe hemorrhagic form [39, 40], the early definition of these characteristics can improve management efficiency. Thus, in order to guarantee maintenance of parameters throughout the 2007 Marilia outbreak, the patients were divided into four temporally consecutive subsets for comparative analysis. The results indicated temporal homogeneity in demographic and hematological profiles, especially brought into evidence by logistic regression and ROC curves (Table 3 and Fig. 2). This showed LEU and PLA |to be independent factors associated to DENV serological positive diagnosis during the whole period.

There was also a statistically significant association of hematological variables in all DENV serological positive samples (Table 5), compatible with DENV infection laboratory findings [37]. However, certain differences were found by additive logistic regression analysis, when comparing the entire dataset and subsets s1 to s4 (Tables 4 and 6). The most significant difference occurred in subset s3, which presented high correlations with DENV positive serological diagnosis. This difference could be attributed to specific unidentified features related to sampling during the period, since this was the only statistical significant variable to be associated to DENV diagnosis.

Currently, methods for the specific treatment and effective prevention of DENV infection are still unavailable. Furthermore, a specific and sensitive method for early DENV-diagnosis is not accessible to all the regions and patients during the course of long dengue epidemics in developing countries, this including Brazil. In order to improve dengue management in this situation, an alternative strategy used by public health care institutions in São Paulo State is, according to the size of the population, determine an epidemiological threshold after the epidemic has reached the incidence rate. Before this, patients undergo dengue laboratory diagnosis. Considering our results, if the epidemiological and hematological profile patterns of the dengue-affected part of the population are investigated in the beginning of the outbreak, when specific diagnosis is carried out after the threshold is reached, the resultant hematological profiles could be used with more accuracy to improve disease management. Furthermore, to reduce the number of dengue false-positives from exclusive clinical-criteria diagnosis, individuals notified as being infected but presenting high scores for LEU and PLA, could be submitted to dengue serological testing. Thus, definition of the epidemiological pattern of DENV transmission in regions with recurrent outbreaks could help healthcare institutions to prevent virus spread and manage associated diseases.

## Conclusions

Herein it was shown that at the outbreak, in a middle-sized town of São Paulo State, Brazil, the clinical criteria applied after an epidemiological threshold has been reached was efficient in managing both classical and more severe cases of DF, since more than 70 % of the reported cases had been confirmed by serologic detection of the DENV NS1 antigen, associated to low leukocyte and platelet counts. The epidemiological pattern of DENV transmission in the town of Marilia was similar to the American prototype, with a prevalence of DF in the adult population and the low occurrence of hemorrhagic forms (0.4 %). Furthermore, demographic and hematological profiles showed a conservative temporal pattern which points to the possibility of making use of the characteristics of an outbreak detected early in the beginning, as a means of improving management throughout all the remaining period. Furthermore, public healthcare institutions could be instructed on how to inform any change in the specific pattern of the outbreak, and thus facilitate investigation of the possible simultaneous occurrence of a new disease, as well the opportune local entry of a different circulating DENV genotype.

## Abbreviations

AGE, age; AIC, akaike information criteria; AL%, percentage of atypical lymphocytes; ALT, alanine aminotransferase; AST, aspartate aminotransferase; C, capsid; DENV, dengue virus; DF, dengue fever; DHF, dengue hemohrragic fever; DSS, dengue shock syndrome; E, envelope; LEU, leukocyte; NS, non-structural protein; PLA, platelets; prM, pre-membrane; ROC, receiver operating characteristic; SEX, gender; SGOT, serum oxaloacetic transaminase; SGPT, serum glutamic pyruvic transaminase

## Additional file

Additional file 1: Multilingual abstracts in the five official working languages of the United Nations. (PDF 386 kb)
