# Supplementary material for: Maintenance of demographic and hematological profiles in a long-lasting dengue fever outbreak: implications for management
Source: Infect Dis Poverty. 2016 Sep 5;5(1):84. doi: 10.1186/s40249-016-0177-y (PMC5011355; doi:10.1186/s40249-016-0177-y)
Supplement: Additional file 1: — Multilingual abstracts in the five official working languages of the United Nations. (PDF 386 kb) [file 40249_2016_177_MOESM1_ESM.pdf]

Translation of the abstract into the five official working languages of the United Nations

الإدارة على الم ترت بة الآث ار :الأمد طوي لة ال ضنك حمى ت فشي أث ناء والدموية الدي مغراف ية الملامح على الح فاظ

أندري ,[1]ري ك ينا ماركوندي س م يشيل ,[1,3]سوزوكي ب وزي نارو دريجو روناء ,[1,2]كارمو ساذ توس دوس موري را أندري ا
[1*] س ب يراذ كا أب اري ث يدا ومار س يا [4]إي تروف ي تش

ت لخ يص

ب عض وف ي .الم عي نة الإق ل يم ية وال سريرية الوب ائ ية الخ صائ ص حاليًا (DF) ال ضنك حمى ت فشي ي م ثل :**عامة معلومات**
/ حالة 150 وقوع إلى ال توصل وب عد ب ال برازي ل، ب اولو، ساو ولاي ة ف ي (ن سمة 000 250-000 100) الحجم م تو سطة المدن
خلال .(DENV) ال ضنك حمى ف يروس عدوى وجود إلى ال سريري ال تشخ يص أ شار ،("الوب ائ ية ال ع ت بة") من ن سمة 000 100
أعرا ضها ت توافق ال تي الأمراض ت لك ال ضنك حمى غ ير موسمياً الم عدي ة الأمراض من أخرى أمراض ظهور وي مكن ,ال ف ترة هذه
عل يه ي ترت ب ما ب المرضى ال صحية ال رعاية مرافق اك تظاظ مع وذلك ل لحمى، المحاك ية ال جسدية وال علامات الأعراض مع
ت جنب ف ي الم صل ية الاخ ت بارات خلال من ل لذج ية ال سريري ال تشخ يص ت أك يد ي ساعد وق د .رئ يسة اذ تكا سات
وم تلازمة (DHF) ال نزف ية ال ضنك ب حمى ب عد ف يما ل لإصاب ة عرضة ي كونوا قد الذين المرضى لدى ال خاطئ ال تشخ يص
ال خصائ ص عن ال كشف ف ي م ف يدة هي ل لمرضى وال دموية ال ديموغراف ية الملامح ف إن ,ذلك على علاوة .(DSS) ال ضنك صدمة
.ال ضنك صدمة وم تلازمة ال نزف ية ال ضنك وحمى ال ضنك ب حمى ةالمرت بط المحددة الأول ى
ب أنهم ف قط ب اولو ساو ولاي ة غرب شمال ف ي ماري ل يا من مريضًا 456 ت شخ يص جرى ،2007 ي ون يو ح تى مارس ومنذ **:الطرق**
لم س تضدات (NS1) 1 اله يكلي غ ير الدم مصل لاخ ت بار خضعوا كما ال سريرية، المعاي ير ح سب ال ضنك ب حمى م صاب ين
(وال عمر ال جنس) ال ديموغراف ية ل لملامح وفقًا الم قارن ال تحل يل ف ي ال فردية ال ن تائ ج ا س تخدام وجرى .ال ضنك روس ف ي
الزمن ية الأن ماط ت ق ي يم جرى كما .(ال نمط ية غ ير ال ل يم فاوية الخلاي ا ون س بة وال ص ف يحات، ال ب يض ال كري ات) الدم وأمراض
ب عدوى ت ن بؤ عوامل ب و ص فها الم سجلة الم تغ يرات ب ا س تخدام وذلك ،الأول ي ال حضور ل زمن وفقًا ال ب يان ات ت ق سيم خلال من
.ROC ومنحن يات ال لوج س تي الان حدار نماذج ف ي ال ضنك ف يروس
وال ص ف يحات ال ب يضاء ال كري ات وكان ت .المرضى من %70.6 لدى موجبًا الم صلي ال ضنك ف يروس اك ت شاف وكان **:ال ن تائ ج**
= 3 + ال ضنك ف يروس ل كل الم توال ية الم تو سطات) ال ضنك ف يروس ب عدوى ال ت ن بؤ ف ي أهم ية الأك ثر ال عوامل هي الأق ل
223 = -ال ضنك وف يروس مل / خل ية 896 134 = + ال ضنك وف يروس مل، / خل ية 760 6 = -ال ضنك وف يروس مل / خل ية 715
ال ت فشي رغم ت طوال مؤق ت حماي ة نمط والدموي ة ال ديموغراف ية الملامح جم يع ق دمت ف قد ذلك، على وعلاوة .(مل / خل ية 872
.الأمد طوي لة
أجل من مفيدًا هذا وكان م بكرة، مراحل خلال ال حماي ة نمط ت حدي د إمكان ية الوب اء ف ترة طوال الات ساق ي سر كما **:الا س ت ن تاجات**
.الم ت ب ق ية ال ف ترة خلال الإدارة ت ح سين

Translated from English version into Arabic by Fathia Sobhi, through

TRANSLATORS
WITHOUT BORDERS

持久的登革热暴发中人口统计学与血液细胞学概况的稳定对疫情管理的启示

Andréia Moreira dos Santos Carmo[1,2], Rodrigo Buzinaro Suzuki[1,3], Michele Marcondes Riquena[1], André Eterovic[4] and Márcia Aparecida Sperança[1*]

摘要:

引言：登革热暴发通常表现出地方性的特异性流行病学与临床特征。在巴西圣保罗的某些中型城市（100 000-250 000居住人口）中，居民的感染率达到万分之十五（150 /100 000）的流行病学阈值，并临床诊断为登革热病毒感染时，其他症状与临床表现与登革热相似的季节性传染病也会同时发生，从而使卫生保健机构过度拥挤。应用血清学检测进行登革热临床确诊能有助于减少患者的误诊，有些患者可能会出现登革出血热和登革热休克综合征。此外，患者的人口统计学和血液细胞学有助于发现与登革热、登革出血热和登革热休克综合征相关的特异性早期特征。

方法： 对圣保罗西北城市马里利亚于2007年三月至六月仅由临床诊断标准诊断的456例登革热患者进行非结构蛋白1（non-structural 1，NS1）登革热病毒抗原检测，并根据人口统计学（性别、年龄）和血液细胞学（白细胞和血小板计数、不规则淋巴细胞比例）情况将结果用于比较分析。同时根据首次出现的时间将数据分割，把记录变量作为logistic回归和ROC曲线中登革热病毒感染的预测因子，对时序模式进行评价。

结果： 456人中有70.6%经血清学检测为阳性。白细胞和血小板数量减少是预测该病毒感染最重要的因素（白细胞中位数是：DENV+ = 3 715个/ml和DENV- = 6 760个/ml, 血小板中位数：DENV+ = 134 896个/ml和DENV- = 223 872个/ml）。此外，所有人口统计学和血液细胞学概况体现了该次长期的暴发处于一种保守的时序模式。

结论：疫情的稳定有利于定义疫情早期的保守模式，这些对改善后期疫情的管理具有重要意义。

Translated from English version into Chinese by Yin Jianhai, through

CHINESE CENTER FOR DISEASE CONTROL AND PREVENTION
NATIONAL INSTITUTE OF PARASITIC DISEASES

Administration de profils démographiques et hématologiques dans le cadre d'une épidémie prolongée de dengue : implications pour la gestion

Andréia Moreira dos Santos Carmo[1,2], Rodrigo Buzinaro Suzuki[1,3], Michele Marcondes Riquena[1], André Eterovic[4] et Márcia Aparecida Sperança[1*]

Résumé

Contexte : les épidémies de dengue présentent des caractéristiques épidémiologiques et cliniques spécifiques du point de vue régional. Le diagnostic clinique a indiqué une infection par le virus de la dengue (DENV) dans certaines villes moyennes (100 000 à 250 000 habitants) de l'État de São Paulo (Brésil) et après le dépassement d'un taux d'incidence de 150 cas/100 000 habitants (« seuil épidémiologique »). Au cours de cette période, d'autres maladies infectieuses saisonnières présentant des symptômes et des signes physiques similaires à la dengue peuvent simultanément apparaître et provoquer par conséquent une surcharge des établissements de soins de santé, constituant ainsi un problème majeur. La confirmation du diagnostic clinique de la dengue à l'aide de tests sérologiques peut contribuer à la prévention des erreurs de diagnostic chez des patients qui risquent ultérieurement de souffrir de dengue hémorragique (DHF) et de dengue avec syndrome de choc (DSS). De plus, les profils démographiques et hématologiques de patients s'avèrent utiles pour détecter des caractéristiques précoces spécifiques associées à la dengue, à la DHF et au DSS.

Méthodes : de mars à juin 2007, 456 patients de Marilia dans le nord-ouest de l'État de São Paulo chez lesquels la dengue avait uniquement été diagnostiquée sur la base de critères cliniques ont subi un test sérologique de dépistage d'antigènes DENV non structurels 1 (NS1). Des résultats individuels ont été utilisés dans le cadre d'une analyse comparative en fonction de profils démographiques (sexe, âge) et hématologiques (numération des leucocytes et des

plaquettes, pourcentage de lymphocytes atypiques). Des modèles temporels ont été évalués en subdivisant les données en fonction du moment de la consultation initiale à l'aide de variables enregistrées, tels que les prédicteurs d'infection DENV dans des modèles de régression logistique et sur des courbes CRO.

Résultats : la détection sérologique du DENV était positive dans 70,6 % des patients. La présence de faibles numérations de leucocytes et de plaquettes constituait le principal facteur de prédiction d'une infection DENV (médianes respectives : DENV+ = 3715 cellules/ml et DENV- = 6760 cellules/ml, et DENV+ = 134 896 cellules/ml et DENV- = 223 872 cellules/ml). De plus, l'ensemble des profils démographiques et hématologiques présentaient un modèle temporel conservateur tout au long de cette épidémie prolongée.

Conclusions : la cohérence obtenue au cours de l'épidémie ayant facilité la définition du modèle de conservation tout au long des étapes précoces, cette approche s'est avérée utile à l'amélioration de la prise en charge au cours de la période restante.

Translated from English version into French by eric ragu, through

TRANSLATORS
WITHOUT BORDERS

Корректирование демографического и гематологического профилей при продолжительной вспышке лихорадки денге: заключения для последующего управления процессом

Андрейа Морейра дос Сантос Кармо[1,2], Родриго Бузинаро Сузуки[1,3], Микеле Маркондес Рикена[1], Андре Этерович[4] и Марсия Апаресида Сперанса[1*].

Тезисы

Историческая справка: Вспышки лихорадки денге (DF) представляют эпидемиологические и клинические характеристики, обладающие региональными особенностями. В некоторых городах среднего размера (100000-250000 жителей) штата Сан Паоло, Бразилия после достижения 150 случаев заболевания на 100000 человек («эпидемиологический порог»), клиническая диагностика выявила инфекцию вируса денге (DENV). На протяжении этого времени могут одновременно появиться другие сезонные инфекционные заболевания с симптомами и физическими признаками сходными с DF. Основной сложностью в таких случаях становится переполнение лечебных центров и больниц. Подтверждение клинического диагноза DF с помощью серологических анализов может помочь избежать ложного диагностирования пациентов, которые могут позже переносить геморрагическую форму лихорадки денге (DHF) и синдром шока денге (DSS). К тому же, демографический и гематологический профили пациента являются полезными при выявлении особых ранних признаков, связанных с DF, DHF и DSS.

Методы: С марта по июль 2007 года 456 пациентов из Марилии на северо-западе штата Сан Паоло, которые были диагностированы только с DF по клиническим признакам прошли серологический анализ на неструктурные антигены 1 DENV (NS1). Индивидуальные результаты были использованы в сравнительном анализе в соответствии с демографическими (пол, возраст) и гематологическими (количество лейкоцитов и тромбоцитов, процент атипичных лимфоцитов) профилями. Временные структуры были проанализированы с помощью подразделения данных в соответствии со временем первоначального посещения пациента,

используя зафиксированные переменные в качестве показателей инфекции DENV в регрессионных логистических моделях и кривых ROC.

Результаты: Серологическое выявление DENV оказалось положительным в 70,6% случаях. Малое количество лейкоцитов и тромбоцитов было самым важным фактором в предположении инфекции DENV (соответствующие медианы DENV+ = 3715 клеток/мл и DENV- = 6760 клеток/мл, и DENV+ = 134 896 клеток/мл и DENV- = 223 872 клеток/мл.) Вдобавок, все демографические и гематологические профили представили консервативную временную структуру на протяжении этой долговременной вспышки.

Выводы: Так как стабильность на протяжении всего периода эпидемии облегчила определение консервативной структуры на ранних стадиях, то это явилось полезным для улучшения управления процессом в течение оставшегося периода времени.

Translated from English version into Russian by Anna Haas, through

TRANSLATORS WITHOUT BORDERS

Mantenimiento de perfiles demográficos y hematológicos en un brote de dengue de larga duración: implicaciones para su manejo

Andréia Moreira dos Santos Carmo[1,2], Rodrigo Buzinaro Suzuki[1,3], Michele Marcondes Riquena[1], André Eterovic[4] y Márcia Aparecida Sperança[1*]

Resumen

Antecedentes: Los brotes de fiebre del dengue presentan características epidemiológicas y clínicas regionalmente específicas. En ciertas ciudades medianas (100.000 a 250.000 habitantes) del estado de São Paulo, en Brasil, y luego de llegar a una incidencia de 150 casos por cada 100.000 habitantes ("marco epidemiológico"], el diagnóstico clínico indicó una infección por el virus del dengue (DENV). Durante este período, otras enfermedades infecciosas estacionales con síntomas y signos físicos que imitaban los de la fiebre del dengue, pueden ocurrir simultáneamente, y la principal desventaja es la resultante sobrecarga de los centros sanitarios. La confirmación del diagnóstico clínico de fiebre del dengue mediante pruebas serológicas puede ayudar a evitar los diagnósticos erróneos en pacientes que podrían luego desarrollar fiebre del dengue hemorrágico y síndrome de choque por dengue (SCD). Además, los perfiles demográficos y hematológicos de los pacientes son útiles para la detección de características tempranas específicas asociadas con la fiebre del dengue, la fiebre del dengue hemorrágico y el síndrome de choque por dengue.

Métodos: De marzo a junio del año 2007, 456 pacientes de Marilia en el noroeste del estado de São Paulo que solo habían sido diagnosticados con fiebre del dengue por criterios clínicos, se sometieron a pruebas serológicas para antígenos no estructurales 1 del virus del dengue (NS1). Los resultados individuales se utilizaron en un análisis comparativo de acuerdo a los perfiles demográficos (género, edad) y hematológicos (recuento de leucocitos y plaquetas, porcentaje de linfocitos atípicos). Los patrones temporales se evaluaron mediante subdivisión de la información de acuerdo a la fecha de atención inicial, utilizando variables registradas como indicadores de infección por el virus del dengue en modelos de regresión logística y curvas ROC.

Resultados: La detección serológica del virus del dengue fue positiva en el 70,6% de los pacientes. La disminución en el recuento de leucocitos y plaquetas fue el factor más importante en la predicción de la infección por el virus del

dengue (media respectivas DENV+=3715 células/ml y DENV-= 6760 células/ml, y DENV+= 134896 células/ml y DENV-= 223872 células/ml). Además, todos los perfiles demográficos y hematológicos presentaron un patrón temporal conservador a lo largo de este brote de larga duración.

Conclusiones: La consistencia a lo largo de la epidemia facilitó la definición de un patrón conservador durante las etapas tempranas, lo que fue útil para mejorar el manejo durante el resto del período.

Translated from English version into Spanish by Maria Alejandra Aguada, through

TRANSLATORS
WITHOUT BORDERS
